# A binding question: the evolution of the receptor concept

**DOI:** 10.1016/j.endeavour.2009.09.001

**Published:** 2009-12

**Authors:** Andreas-Holger Maehle

**Affiliations:** Centre for the History of Medicine and Disease, Wolfson Research Institute, Durham University, Queen's Campus, Stockton TS17 6BH, United Kingdom

## Abstract

In present-day pharmacology and medicine, it is usually taken for granted that cells contain a host of highly specific receptors. These are defined as proteins on or within the cell that bind with specificity to particular drugs, chemical messenger substances or hormones and mediate their effects on the body. However, it is only relatively recently that the notion of drug-specific receptors has become widely accepted, with considerable doubts being expressed about their existence as late as the 1960s. When did the receptor concept emerge, how did it evolve and why did it take so long to become established?

## ‘A beautiful but remote lady’

*To most of the modern pharmacologists the receptor is like a beautiful but remote lady. He has written her many a letter and quite often she has answered the letters. From these answers the pharmacologist has built himself an image of this fair lady. He cannot, however, truly claim ever to have seen her, although one day he may do so*[1].

So wrote Dutch pharmacologist D.K. de Jongh as recently as 1964. For him and others, the cell receptor was something of an enigma. Not least, there was the problem of understanding how cells seemed to boast receptors for manmade chemical substances synthesized in the laboratory. For German professor of pharmacology Klaus Söhring speaking at a colloquium in Hamburg in the early 1950s, this conundrum was so great that it all but ruled out the existence of such specific receptors. After all, he argued, how could God, the Creator, have known which different kinds of pharmaceutical substances would be developed by mankind [2].

Such doubts about receptors were only dispelled with the development of the first receptor-specific remedies, in particular the beta-receptor blockers for the therapy of hypertension and the histamine-H2-receptor blockers for the treatment of stomach ulcers. James Black, who was the originator of both these groups of drugs, was awarded the Nobel Prize for Medicine or Physiology in 1988 [3]. By this time other researchers had elucidated the protein structure and genetic basis of various other receptors and had even visualised some of them with electron microscopy. It became increasingly clear that the human body contained many hundreds of receptor subtypes, opening up a vast new field of research to the pharmaceutical industry. The aim was to target these receptors for the specific treatment of various diseases, ranging from cystic fibrosis to epilepsy [4].

Given the ubiquity of receptors in today's biomedical sciences, it is worth looking into the history of these molecules in more detail. In particular, where did the concept of a receptor come from, why was concrete evidence for its existence so long in coming and what role did the notions of substance binding and of specific effects of substances play in this process?

## Early thinking

**Figure 1:**
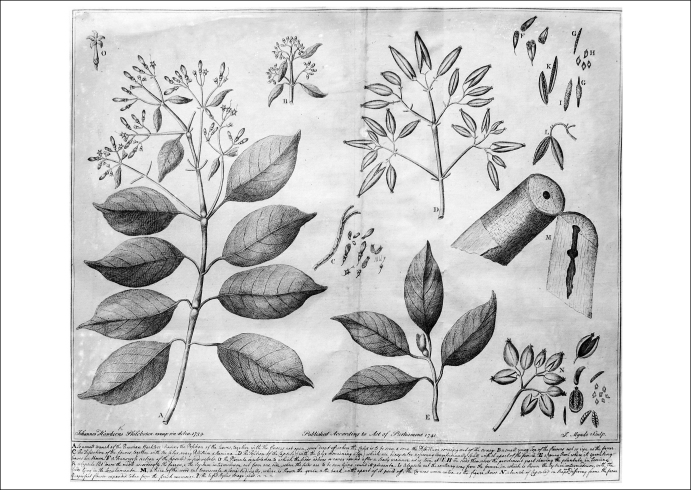
Peruvian bark tree. Engraving by J. Howkins. Wellcome Library, London.

The specificity of certain drugs to particular diseases had been highlighted at least since the seventeenth century. Perhaps the best-known example is the efficacy of Peruvian bark, the predecessor of quinine, in the treatment of intermittent fevers or malaria (Figure 1) [5]. Yet, such specificity remained a mysterious phenomenon. As one sceptical author put it in 1797:

… *as to specifics, if their idea be explicable by supposing an admiral sent down channel, across the Bay of Biscay, and up to the Mediterranean, with express orders to attack the Maltese, but with the strictest charge not to molest any other state whatever; I cannot conceive any medicine such a specific as to conform most punctually with such orders, to act vigorously against one particular gland or humour of the body, without in the least affecting or disturbing any other* … [6].

In the Romantic period, it became more commonplace to encounter discussions of specificity. Writers like the German Friedrich Sobernheim (1803–1846), for example, began to use the physico-chemical concept of elective affinities to rationalize the predilection of certain substances, including the newly isolated alkaloids, to affect particular parts of the body. For Sobernheim, strychnine had a specific affinity to the spinal cord, digitalis and tobacco to the nerves of the heart, alcohol to the brain, mercury to the salivary glands, ergot to the nerves of the uterus, and sulphur to the skin [7]. Moreover, the English physician James Blake (1814–1893) demonstrated in the 1840s that solutions of inorganic compounds with the same macroscopic crystalline structure produced similar physiological effects when infused intravenously into animals. This led to further research on the relationship between the chemical structure and pharmacological effect of substances such as salts and the substitution products of various alkaloids [8].

By the start of the twentieth century, a scientific controversy had developed over whether pharmacological action depended directly on the chemical structure of a substance or rather upon its physical properties [9]. As we shall see, this general problem formed an important background to the subsequent debate about the existence and relevance of receptors.

## Side-chains and receptive substances

**Figure 2:**
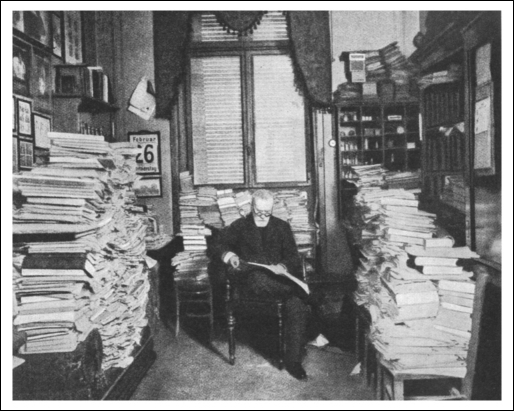
Paul Ehrlich in his study. Reproduced from de Kruif, P. (1927) Mikrobenjäger. Orell Füssli, Zürich.

**Figure 3:**
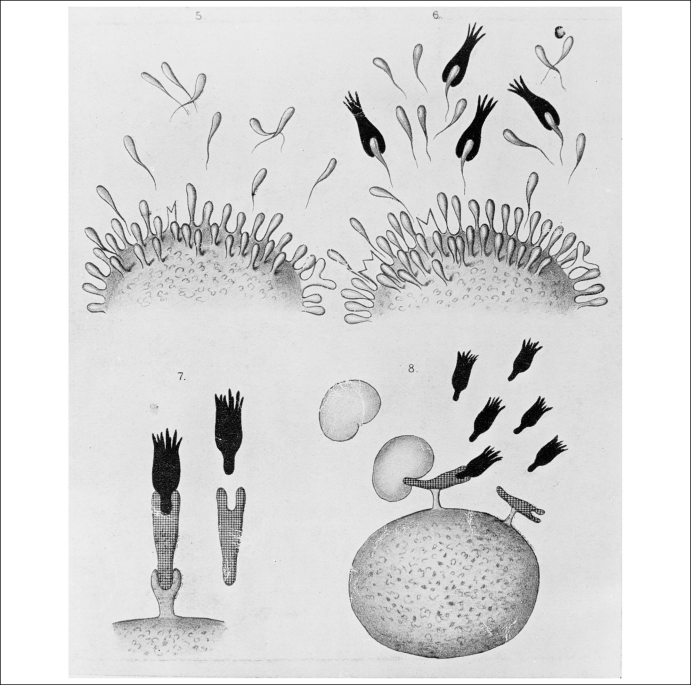
Diagrams illustrating Paul Ehrlich's side-chain theory. Wellcome Library, London.

The role of Paul Ehrlich (1854–1915) in the development of the receptor concept is quite well-known [10], and I will therefore only briefly sketch it here (Figure 2). Ehrlich's side-chain-theory formed an important basis for his work on blood cells and on chemotherapy of infectious diseases such as sleeping-sickness and syphilis. In 1910, his research led to the introduction of Salvarsan, the famous ‘magic bullet’ against the germs of syphilis. Ehrlich's side-chain-theory was developed in the course of his studies into the staining of body cells and tissues, into the oxygen consumption of cells, and especially on the interaction between bacterial toxins and the so-called anti-toxins or antibodies formed by the body. In 1897 Ehrlich published for the first time a full account of his side-chain-theory of anti-toxin formation. The large ‘molecule’ of the cell protoplasm was supposed to have certain side-chains that were able to bind chemically the toxins produced by the bacteria. The thus occupied side-chains became unable to fulfil their usual functions in nutrition and oxygen consumption, forcing the cell to produce more side-chains. A surplus of side-chains was released into the blood stream where they bound as anti-toxins or antibodies to the bacterial toxins—forming thus the basis of immunity (Figure 3). In 1900 Ehrlich replaced the term side-chain (or *Seitenkette*) with the term *Receptor* [11].

**Figure 4:**
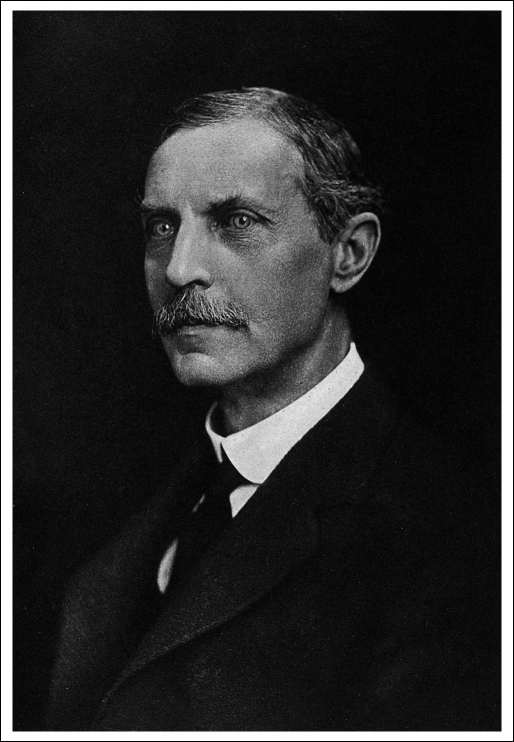
John Newport Langley. Photogravure. Wellcome Library, London.

However, Ehrlich initially believed that receptors existed only for toxins and for physiological foodstuffs and ferments. Drugs and medicines could quite easily be washed out of body tissues with solvents, so that he did not assume that they were fixed to specific components of the cell. He changed his mind only in 1907 [12], partly due to results of his own further research, but in particular also because of a different kind of receptor theory that had been proposed by the Cambridge physiologist John Newport Langley (1852–1925) (Figure 4). Langley's concept is particularly interesting because it placed special importance on the notion of substance and of substance binding.

In the mid-1870s, as a student of Michael Foster (1836–1907), Langley had been given the task of studying the effects of the plant drug jaborandi in animals, in particular its effect on the heart. Foster was interested in whether the automatic activity of the heart originated from its own muscle fibres or from nerves leading to the heart. Langley showed that the effect of jaborandi – a slowing down of the heart beats – occurred when the heart nerves were paralyzed using curare. He also demonstrated that the effect of jaborandi could be reversed by dripping a solution of atropine directly on the exposed animal heart. Both these findings indicated that the drug jaborandi acted directly on the heart muscle and, to a certain extent, supported Foster's view that the automatic heart beat probably originated from within the heart muscle itself [13].

This led Langley to pursue two lines of research that were also of interest to other physiologists of the time: one was the question whether pharmacological substances act directly on the body tissues or indirectly by affecting the endings of nerves leading into these tissues; the other was the question how antagonistic action between drugs, for example the one Langley had shown between jaborandi and atropine, came about. In a further series of experiments, he demonstrated the antagonism between pilocarpine (an alkaloid of jaborandi) and atropine on salivary secretion in dogs and cats; pilocarpine stimulated secretion, atropine stopped it, a new dose of pilocarpine got it going again, a further dose of atropine stopped it, and so on. How could this be explained? Obviously the relative concentration of the two substances in the animal body played a role, and both substances had to have a chemical affinity to the relevant tissues. In 1878 Langley formulated the following hypothesis:

… *we may, I think, without much rashness, assume that there is some substance or substances in the nerve endings or gland cells with which atropin and pilocarpin are capable of forming compounds. On this assumption then the atropin or pilocarpin compounds are formed according to some law of which their relative mass and chemical affinity for the substances are factors. In the analogous case with inorganic substances, other things being equal, these are the sole factors. To take the simplest case, if **a** and **b** are both able to form, with **y**, the compounds **ay**, **by**, then **ay** and **by** are both formed, quantity of **ay** and **by** depending on the relative masses of **a** and **b** present and their relative chemical affinity to **y***[14].

In other words, Langley used the analogy between two inorganic chemical substances competing for reaction with the same third inorganic substance as a model to explain antagonistic drug action in the body. This implied that the relevant body cells contained some specific substances to which the drug substances had a chemical affinity. This thought, as we shall see, formed the basis of his later receptor concept.

Langley formulated his receptor concept only about 30 years later, because his research path led him into other areas, in particular into the physiology of the autonomic nervous system, a field in which he became an international authority. However, his neurophysiological research led him eventually back to the question of drug antagonisms.

The key experiment for his receptor concept involved the antagonism between nicotine and curare and was carried out on an anaesthetized rooster. Injection of nicotine led to a characteristic contraction of certain muscles of the leg, recognizable in the stiff, extended legs of the animal. This effect could be antagonised by injecting curare, resulting in the relaxation of the leg muscles. This antagonism could also be shown if the relevant nerves of the leg muscles had been cut through and allowed to degenerate, which meant for Langley that the two drugs acted on the muscle tissue directly. Just like pilocarpine and atropine, nicotine and curare competed for the same substances in the protoplasm of the cells. Moreover, after application of curare the relaxed leg muscles could be made to contract by applying an electric current. Langley concluded from this finding that neither the drugs nor the electric stimulus acted directly on the contractile substance of the muscle cells, but on what he called ‘accessory substances’. And, he continued, ‘Since this accessory substance is the recipient of stimuli which it transfers to the contractile material, we may speak of it as the *receptive substance* of the muscle [15]’.

This statement, made in 1905, was the first clear formulation of Langley's receptor concept. He was very quick to generalize it, suggesting that it could be applied to explain also the action of other alkaloids, such as pilocarpine, atropine and strychnine, and of hormones, such as adrenalin, secretin, thyroidin and the sex hormones. And he wrote in general terms:

*So we may suppose that in all cells two constituents at least are to be distinguished, a chief substance, which is concerned with the chief function of the cell as contraction and secretion, and receptive substances which are acted upon by chemical bodies and in certain cases by nervous stimuli. The receptive substance affects or is capable of affecting the metabolism of the chief substance*[16].

Langley was aware of the similarity between his concept of receptive substances in cells and Paul Ehrlich's side-chain theory. However, he maintained that they had uncovered somewhat different phenomena. Both assumed, as he put it, ‘atom-groups of the protoplasm’ of the cell, to which substances could bind. But while Ehrlich's side-chains were ‘fundamental’ to the cell's life (i.e. the cell would die if they were all occupied by poisons), Langley's ‘receptive substance’ merely modified the cell's function when a drug or hormone bound to it. Significantly, Langley never adopted Ehrlich's more general term ‘receptor’, but stuck to his own term ‘receptive substances’ throughout his life. Ehrlich, on the other hand, conceded that the receptor concept was also applicable to drugs and medicines, not only to toxins or foodstuffs.

## The potential-poison theory

Both Ehrlich and Langley had conceptualised the ‘receptor’ through analogy with chemical binding of substances, and their theories were viewed as ‘chemical’ in nature. This, however, meant that their theories got caught up in the controversy over whether pharmacological substances acted primarily through their chemical or their physical properties. The great competitor to the receptor concepts of Ehrlich and Langley was the so-called ‘potential-poison’ theory of the German pharmacologist Walther Straub (1874–1944).

Straub had developed it during a research stay at the *Stazione Zoologica* in Naples at the start of the new century. There, he studied the antagonism between atropine and the poison of the fly agaric, muscarine, on the heart of the sea snail (*Aplysia)* and the torpedo fish*.* In essence, he argued that a poison acted as long as there was a concentration difference or ‘potential’ between the outside and the inside of the cell. The effect was due to a deformation or other physical disturbance of the cell membrane when the poison molecules penetrated it. Like Langley, Straub was quick to generalize, suggesting that his physical theory of drug action applied also to other alkaloids, such as pilocarpine, physostigmine and nicotine, and to the hormone adrenalin [17]. Straub's theory found significant supporters in Britain, in particular Henry Dale (1875–1968) and George Barger (1878–1939) of the Wellcome Physiological Research Laboratories and Arthur Cushny (1866–1926), who held the chair in pharmacology at University College London [18].

Straub offered a direct challenge to Ehrlich's theory of so-called chemoreceptors. At the annual assembly of German naturalists and doctors in 1912, Straub pointed out that there were many pharmacologically active substances (such as nitrous oxide, carbonic acids and kali salts) whose constitution made it very unlikely that they were capable of reacting chemically within the organism. There might be receptors for certain poisons, he conceded, but to build a whole theory of chemoreceptors on this was impermissible [19].

Moreover, it seems that Straub did not fully accept Langley's work as a contribution to the field of pharmacology. As late as 1938, Straub addressed the International Congress of Physiologists in Zurich with the following statement:

… *I may perhaps remind you that there are two types of pharmacologist: those who study the living organism with a chemical substance, for example **Claude Bernard** with curare or **Langley** with nicotine, and such who use a living organism to study a chemical substance; the former practise physiology, the latter pharmacology*[20]!

## A receptive audience

However, the interwar period had also brought two important supporters of Langley's theory of receptive substances and of receptor theory more generally on the scene. Alfred Joseph Clark (1885–1941), who succeeded Cushny in the chair at UCL in 1920 and again, after Cushny's death in 1926, in the Edinburgh chair of Materia Medica; and John Henry Gaddum (1900–1965), who became professor of pharmacology at UCL in 1935. Both Clark and Gaddum had been students of Langley in Cambridge.

Based on quantitative dose-effect studies with the transmitter substance acetylcholine, Clark suggested the so-called receptor occupancy theory. According to this theory the intensity of the pharmacological effect of a substance was directly proportional to the number of cell receptors occupied by the substance [21]. Gaddum, working on the dose-effect relations of adrenalin and ergotamine elucidated the competitive antagonism between two substances at receptors and introduced the notion of receptor blockage by the antagonistic substance [22]. Clark publicly attacked Straub over his physical potential-poison theory, saying at a meeting of the Royal Society in London in 1936 that it assumed processes that were unknown in physical chemistry [23].

Despite such support and development of the receptor concept, the critics remained vocal. Important was the position of Sir Henry Dale, who was awarded the Nobel Prize for his neurotransmitter research in 1936. Dale was much more interested in the transmitter substances in the nervous system than in their potential receptors. As late as 1943, at a conference of the Faraday Society in London, he called the worth of the receptor concept for explaining specific drug action into question:

*It is a mere statement of fact to say that the action of adrenaline picks out certain such effector-cells and leaves others unaffected; it is a simple deduction that the affected cells have a special affinity of some kind for adrenaline; but I doubt whether the attribution to such cells of “adrenaline-receptors” does more than re-state this deduction in another form*[24].

When, 5 years later, the American pharmacologist Raymond P. Ahlquist (1914–1983) proposed the existence of two types of adrenaline-receptors, alpha and beta, mediating different patterns of pharmacological action, his paper on this topic was rejected by the *Journal of Pharmacology and Experimental Therapeutics*. Through personal contacts with the editor of the *American Journal of Physiology* he eventually managed to get it published [25]. Ironically, Ahlquist's distinction between the two types of adrenaline-receptors became the basis for the development of the first therapeutically useful receptor blocking drug, the beta-blocker propranolol, which was introduced by (Sir) James Black in 1965. Only then did most pharmacologists start to believe that receptors were more than hypothetical entities, or as de Jongh had called them, ‘beautiful but remote ladies’.

## Conclusion

The concept of ‘receptive substances’ or ‘receptors’ arose from older notions of specific drugs and of elective affinities in the late nineteenth and early twentieth centuries within two different contexts: in immunology (with Paul Ehrlich) and in neurophysiology (with John Newport Langley). Both used the analogy of chemical binding between substances to explain the biological phenomena they studied. This chemical analogy implied the existence of specific substances or molecules in body cells that fixed biologically active substances, such as plant alkaloids, bacterial toxins, hormones and transmitter substances. The chemical character of the receptor concept led to controversy with those who favoured physical explanations of drug action. This conflict continued in the light of merely indirect evidence for about 60 years until the receptor concept led to tangible therapeutic consequences and receptors began to be identified with specific proteins embedded in cell membranes or inside the cells. The multitude of receptors that is now thought to exist provides a great challenge but also significant opportunities for the development of new specific treatments.
